# Challenges Related to the Implementation of an EMS-Administered, Large Vessel Occlusion Stroke Score

**DOI:** 10.5811/westjem.2019.9.43127

**Published:** 2019-10-21

**Authors:** Benjamin J. Lawner, Kelly Szabo, Jonathan Daly, Krista Foster, Philip McCoy, David Poliner, Matthew Poremba, Philip S. Nawrocki, Rahul Rahangdale

**Affiliations:** * Allegheny General Hospital, Department of Emergency Medicine, Pittsburgh, Pennsylvania; † Temple University School of Medicine, Department of Emergency Medicine, Philadelphia, Pennsylvania; ‡ University of Pittsburgh, Joseph M Katz Graduate School of Business, Pittsburgh, Pennsylvania; § Penn Medicine, Division of Traumatology, Surgical Critical Care, and Emergency Surgery, Department of Surgery, Philadelphia, Pennsylvania; ¶ University of Minnesota School of Medicine, Department of Neurology, Minneapolis, Minnesota

## Abstract

**Introduction:**

There is considerable interest in triaging victims of large vessel occlusion (LVO) strokes to comprehensive stroke centers. Timely access to interventional therapy has been linked to improved stroke outcomes. Accurate triage depends upon the use of a validated screening tool in addition to several emergency medical system (EMS)-specific factors. This study examines the integration of a modified Rapid Arterial oCcclusion Evaluation (mRACE) score into an existing stroke treatment protocol.

**Methods:**

We performed a retrospective review of EMS and hospital charts of patients transported to a single comprehensive stroke center. Adult patients with an EMS provider impression of “stroke/TIA,” “CVA,” or “neurological problem” were included for analysis. EMS protocols mandated the use of the Cincinnati Prehospital Stroke Score (CPSS). The novel protocol authorized the use of the mRACE score to identify candidates for triage directly to the comprehensive stroke center. We calculated specificity and sensitivity for various stroke screens (CPSS and a mRACE exam) for the detection of LVO stroke. The score’s metrics were evaluated as a surrogate marker for a successful EMS triage protocol.

**Results:**

We included 312 prehospital charts in the final analysis. The CPSS score exhibited reliable sensitivity at 85%. Specificity of CPSS for an LVO was calculated at 73%. For an mRACE score of five or greater, the sensitivity was 25%. Specificity for mRACE was calculated at 75%. The positive predictive value of the mRACE score for an LVO was estimated at 12.50%.

**Conclusion:**

In this retrospective study of patients triaged to a single comprehensive stroke center, the addition of an LVO-specific screening tool failed to improve accuracy. Reliable triage of LVO strokes in the prehospital setting is a challenging task. In addition to statistical performance of a particular stroke score, a successful EMS protocol should consider system-based factors such as provider education and training. Study limitations can inform future iterations of LVO triage protocols.

## INTRODUCTION

Emergency medical services (EMS) systems are regularly tasked with the delivery of time-sensitive care. Similar to ST-elevation myocardial infarctions, burns, and traumatic emergencies, a major component of stroke-centric care involves the transport of eligible patients to designated centers. The 2018 American Heart Association and American Stroke Association guidelines for acute ischemic stroke recommend a regional system of stroke care that involves rapid identification, diagnostic protocols, thrombolytic medications, and mechanical clot retrieval.^1^ Recently, there has been significant interest with respect to the early identification of large vessel occlusion (LVO) strokes.^2^–^4^ The ability to reliably identify LVO in the prehospital setting would permit EMS providers to preferentially transport patients to comprehensive stroke centers capable of interventional procedures.

Benefits associated with this type of triage strategy include a reduction in secondary transfers and a reduced time to groin puncture when endovascular treatment is pursued.^5^ Current literature suggests that the window of opportunity for interventional stroke therapy may extend well beyond the window for systemic thrombolysis.^6^,^7^ However, there is controversy over how EMS systems operationalize the identification of an LVO. Complicating the situation further, prehospital providers must make this determination rapidly in a chaotic and uncontrolled environment with missing or incomplete information. The goal of identifying LVO strokes is a laudable one, but it assumes that the EMS system can reliably differentiate the patient experiencing an LVO from other stroke syndromes, mimics, or imminent life threats.^8^

In 2016, the Pennsylvania Bureau of EMS approved an optional prehospital protocol that permits providers to use a modified Rapid Arterial oCclusion Evaluation (mRACE) score for the triage of potential LVO patients (Appendix 1). Patients who screened positive would be triaged to a comprehensive stroke center. This retrospective analysis examines the new protocol’s ability to accurately identify patients with suspected LVO stroke and to triage them appropriately to a comprehensive stroke center. Specifically, it was thought that the inclusion of a validated, LVO-specific stroke triage score would improve the prehospital triage process and more accurately identify patients experiencing an LVO. The evaluation of a statewide protocol represents a holistic assessment of a system’s ability to render condition-specific care and involves controversies and challenges beyond the clinical performance of any singular stroke scoring system. Lessons learned from the application of regionalized EMS protocols can inform future efforts and optimize the triage process.

## METHODS

In 2016, the Pennsylvania Bureau of EMS authorized EMS agencies to include an additional stroke assessment into the stroke treatment protocol. The new stroke assessment was applied to patients who screened positive after application of the Cincinnati Prehospital Stroke Score (CPSS). The Pennsylvania mRACE scale was adapted from the original RACE instrument published by Perez de la Ossa in 2014.^4^ Agencies electing to use mRACE had to complete a single, state-approved training module that was delivered via a hybridized (online and didactic) instruction process. Although the class could have been delivered by approved instructors, the EMS bureau authorized a singular curriculum consisting of slides and handouts. Advanced Life Support (ALS) providers, credentialed as a prehospital registered nurse, paramedic, or critical care provider, were authorized to conduct the mRACE examination. Basic Life Support providers could perform the initial stroke screen and request ALS assistance, if appropriate.

**Population Health Research Capsule**

What do we already know about this issue?

Timely and accurate triage of patients with a suspected large vessel occlusion stroke represents a significant diagnostic challenge for EMS providers.

What was the research question?

Does the addition of a modified Rapid Arterial Occlusion Evaluation (mRACE) score to an EMS stroke protocol improve triage accuracy?

What was the major finding of the study?

Implementation of the mRACE score did not contribute to improved triage accuracy of large vessel occlusion strokes.

How does this improve population health?

The study highlights important questions related to systems-based stroke triage. Hopefully, the results will inform future EMS protocols and improve stroke assessment.

A retrospective review of EMS transports to a single comprehensive stroke center in Pennsylvania was performed to identify patients eligible for inclusion. Research assistants from the emergency department performed the first round of chart abstraction. The research coordinator and a chief neurology resident on the stroke service reviewed all charts for agreement with respect to the final diagnosis of LVO. Although mRACE did not appear in the official protocol document until 2017, several EMS agencies were authorized by the bureau of EMS to triage patients in accordance with mRACE guidelines.

Patients transported between November 1, 2016–June 30, 2017 were included in the initial evaluation period. We retrospectively analyzed prehospital charts to search for either a provider impression of stroke or a dispatch category consistent with stroke. Provider impressions included in the analysis consisted of “stroke/TIA,” “CVA,” or “neurological problem.” The retrospective analysis was completed through review and abstraction of the EMSCharts electronic EMS medical health record (emsCharts, Inc; Warrendale, PA) Only patients between the ages of 18–90 with an authorized prehospital stroke score (CPSS or mRACE) were included in the final analysis. Other abstracted data points included the following: EMS call category; patient age and gender; glucose level; electrocardiogram reading; Glasgow Coma Scale (GCS); and vital signs (heart rate, blood pressure, respiratory rate, oxygen saturation). We also collected the final hospital discharge diagnosis for each subject from the discharge summary. The state-approved stroke protocol is available for review in Appendix 1.

The stroke protocol instructs EMS providers to perform a general assessment and then perform the CPSS. Approved providers then conduct the mRACE examination on those patients who tested positive on the initial CPSS. Patients who are assigned an mRACE score of 5 or greater were considered candidates for transport to a comprehensive stroke center. The cutoff score was mandated by the EMS bureau and extrapolated from previous studies involving the original RACE score derivation.^4^,^9^

We compared CPSS and RACE scores to the discharge diagnosis listed in the patient discharge summary. Based on these comparisons we were able to determine the number of patients who falsely tested positive and negative for stroke by EMS providers for both CPSS and mRACE. We also determined the number of patients who were found to be true positive (CPSS- or mRACE-positive with a discharge diagnosis of LVO) and negative (CPSS- or mRACE-negative with a discharge diagnosis other than LVO). Written discharge summaries did not include specific *International Classification of Diseases*, 10^th^ edition, codes. Therefore, the diagnosis of “LVO” was established by the presence of any of the following: 1) anterior cerebral circulation ischemic stroke from a blockage in the anterior cerebral artery, the middle cerebral artery or carotid terminus; 2) posterior cerebral circulation ischemic stroke from a blockage in the posterior cerebral artery or vertebral basilar artery stroke; or 3) endovascular thrombectomy or other interventional radiology procedure targeted at treating a suspected LVO ischemic stroke.

We used these figures to calculate the sensitivity, specificity, positive predictive value, and negative predictive value for each score. “True negative” referred to those individuals who did not have a diagnosis related to acute stroke or LVO upon review of their hospital medical record and discharge summary. We examined secondary variables, including GCS score, vital signs, glucose level, and electrocardiogram findings, for possible trends that could potentially impact the accuracy of CPSS and RACE to identify LVOs in the prehospital setting. Characteristics of the respective stroke scores were used as a surrogate marker for the EMS protocol’s effectiveness. This study was approved by the Allegheny Health Network’s Institutional Review Board.

## RESULTS

The search strategy yielded 380 prehospital charts. Of these, 67 were excluded due to missing or incomplete data leaving 312 for analysis. CPSS was used during 255 patient encounters, mRACE was used on 29 patients, and “other” stroke scales were used on 28 patients encounters. “Other” stroke scales were those not specifically mentioned in the Pennsylvania State EMS protocol. Out of 132 patients who were CPSS positive, 28 false positives were present resulting in a sensitivity of 82% (95% confidence interval [CI], 74.08–88.16). There were 123 CPSS-negative patients including those labeled inconclusive. Twenty-three false negatives occurred in the CPSS group for a calculated specificity of 78%. The positive likelihood ratio for CPSS was calculated at 3.74 (95%CI, 2.67–5.25).

The mRACE score was the second most widely used EMS stroke assessment. The sensitivity of an mRACE score of 5 or greater for LVO was 25% (5% CI, 0.63–80.59). Specificity of the mRACE score for an LVO was calculated at 75% (95% CI, 50.61–87.93). The positive predictive value of mRACE was 12.50 (95% CI, 2.28%–46.61%) and eight out of 29 patients had a positive mRACE score, but only four patients had an LVO. Therefore, the negative predictive value of mRACE > or = to 5 for a LVO was 85.71 (95% CI, 76.41–91.74). EMS providers recorded a blood glucose measurement in a majority (over 73%) of stroke encounters. When provider impression was compared with the initial diagnosis, the most frequently encountered stroke mimic appeared to be seizure or seizure-like activity of various etiologies. Results are summarized in Tables 1 and 2.

## DISCUSSION

Our study represented an initial assessment of a novel, statewide stroke protocol aimed at triaging LVO patients to a comprehensive stroke center. The mRACE score was touted as a valuable tool for the identification of patients who might be appropriate for referral to a regional comprehensive stroke center. State EMS triage protocols instruct prehospital providers to use a single score (mRACE) to make determinations about the presence of an LVO. Because the addition of mRACE into existing treatment protocols represents an evolving process, study authors also examined the ability of the CPSS to identify patients with an LVO. Prior to the rollout of the Pennsylvania mRACE score, the CPSS was the sole instrument used by the region’s EMS providers to confirm a prehospital impression of stroke. Interestingly, the less-discriminatory scale (CPSS) displayed superior sensitivity and specificity for the detection of LVO. Existing literature is replete with various stroke scoring schemes, and system medical directors, managers, and EMS clinicians are tasked with applying the tool that is most appropriate for their system. The challenges associated with prehospital diagnosis paired with the imperative for a rapid, accurate prehospital impression make it exceedingly difficult to come up with a reliable triage algorithm.

Accurate prehospital identification is a crucial step in the appropriate and comprehensive management of acute ischemic stroke. Prehospital providers face many challenges in this task, including limited information and a chaotic and uncontrolled environment, as well as time and resource constraints. Our study shows that the CPSS is the most common prehospital stroke screening tool used within our region. Interestingly, while not specifically validated as an LVO screening tool the CPSS displayed a sensitivity of 88% for the detection of LVO. When EMS providers applied the mRACE tool, we found a 25% sensitivity for LVO. In this preliminary assessment of the stroke triage protocol, the addition of an mRACE score failed to reliably identify those who may benefit from primary transport to a comprehensive stroke center capable of delivering appropriate, interventional-based therapies.

Revolutionary stroke trials starting with MR CLEAN, ESCAPE, REVASCAT, and recently DAWN and DEFUSE-3, have shed light on the utility of extended mechanical thrombectomy for LVO strokes.^10^–^14^ There is increased interest in triaging appropriate patients to centers capable of intervention due to the possibility of improved neurological outcomes and functional recovery. Indeed, a regionalized system of stroke care, which emphasizes validated triage tools and routes patients to centers capable of providing definitive stroke therapy, is essential to achieving the improved outcomes touted in the recent interventional stroke trials.

One of the most important functions of an EMS system is to deliver the patient to the right place, at the right time, and via the correct vehicle. Stroke presents a challenge to EMS providers in that there are many “mimics” that can confound the initial presentation and diagnosis.^2^,^15^ This can make accurate triage of patients experiencing such symptoms challenging. The importance of identifying strokes within a brief time window adds additional pressure to the initial prehospital assessment. Endovascular therapy represents a promising modality for patients suffering from a LVO stroke, and the benefits are proportional to time of therapy delivery.

To reduce the incidence of overtriage, several stroke scoring systems have been developed to assist EMS providers with accurate diagnosis.^16^,^17^ Existing literature affirms that the ideal tool has yet to emerge.^3^,^18^ The Smith (2018) et al. meta-analysis demonstrated that LVO-specific triage schemes failed to perform better than less-selective tools. The Turc (2016) et al. paper examined 13 clinical scores for their ability to predict LVO and observed similar shortcomings with respect to scale accuracy and false positive rates.^18^ EMS systems across the country have experimented with checklists, telemedicine, and other strategies targeted at stroke evaluation.^19^,^20^ Despite a lack of consensus with respect to an optimized stroke triage protocol, current guidelines suggest that EMS systems consider bypassing a primary stroke center in favor of a comprehensive stroke center when LVO is suspected.^1^ However, the added benefits apply only if the EMS system in question can 1) articulate a consistent, accurate protocol for stroke triage, and 2) reliably identify the presence of an LVO.

The literature is replete with analyses of multiple prehospital triage scores. A singular stroke score’s “specificity” or “sensitivity” is a misleading outcome when reported in the absence of a comprehensive and regionalized stroke triage protocol. In other words, the EMS system performing the score is just as important as the score’s accuracy and structure. Apart from a designation of ALS, BLS, or first response, there may be little to no similarity between any two EMS systems. The Pennsylvania Department of Health Bureau of EMS presented EMS agencies with the option of implementing a mRACE score to facilitate accurate triage and transport. The initial rollout of the mRACE score occurred at the discretion of individual medical directors and was predicated upon a review of existing scoring systems. Variabilities in provider familiarity and provider level of education likely contributed to the inconsistent application of mRACE. Despite being designated as the only state-approved scoring system for LVO, the mRACE score was only applied in a small percent of cases.

Since the initial submission of this article, one EMS agency published its “long-term” experience with RACE-based prehospital triage of stroke.^21^ The study included 492 “RACE Alert” patients and boasted a 77% sensitivity for the detection of LVO for scores ≥ to 5. Intracerebral hemorrhage, as opposed to seizure, was the most common stroke mimic found in the intervention group. Paramedics applied the RACE exam to patients scoring positive on CPSS. The study’s promising results highlight important points about protocol formulation and execution. First, the study involved a single EMS agency that benefited from collaboration between medical directors and stroke neurologists. The neurologists were from a single comprehensive stroke center, and all EMS providers in the study were “licensed as paramedics.” RACE training was consistent and uniform; all paramedics had to successfully complete a “four hour module” and undergo annual retraining. The ability to route education and training through one agency likely contributed to the study’s favorable conclusions. In addition, the study’s protocol was restricted to a single group of ALS providers.^21^

Accordingly, the varied composition of our study’s EMS system might also have contributed to the results. EMS agencies in the western Pennsylvania area incorporate volunteer, part-time, and career positions. It logically follows that frequency of exposure to LVO and its clinical manifestations would result in a more nuanced understanding of how to integrate and score clinical findings. Furthermore, the particular EMS region under study does not use a consistent paradigm for medical command. Referring EMS agencies employ a wide range of physician oversight strategies that incorporate anything from episodic physician call review to a more robust physician presence at designated skills-demonstration sessions. Future studies might consider implementing a stroke-scoring scheme within a system that embraces a more consistent mode of physician oversight with respect to both education and quality improvement.

There is a significant disconnect between the specificity and reliability of an LVO triage scheme and its utility within a larger EMS system. Deciding how to operationalize an LVO score into an EMS system requires careful consideration of system-specific factors. Apart from a designated educational program, it is vitally important to identify discrepancies in how the score is applied. EMS provider training and experience may play a significant role in the ability to reliably perform more complex neurological assessments and integrate those findings into an often-undifferentiated clinical picture.^22^ It is hoped that analyses such as this one, although limited in its retrospective approach and single-center design, can shed light on the difficulties implicit in a systemwide application of a stroke triage scheme.

A tried and time-tested scale such as the CPSS holds promise in that it can accurately identify strokes and suggest the presence of LVO.^23^ Richards (2018), et al. examined consecutively enrolled acute stroke patients arriving at a single comprehensive stroke receiving center from 2012–2014. A CPSS score of 3 predicted acute ischemic stroke with a specificity of 88% and a sensitivity of 41%. The unadjusted odds ratio of CPSS for LVO was calculated at 5.1 The authors posited that CPSS could therefore be used as a “screen” for LVO. Reportedly, 72.7% of patients with a CPSS score of 3 were ultimately found to have an LVO. The CPSS score has some significant advantages over other triage scores. Specifically, it is easy to use, requires little to no additional education, and is reproducible between EMS providers.^22^,^24^ Results from this cohort of patients supports the premise of using a high CPSS score as a possible LVO screen.

Although prospectively validated, the RACE score’s generalizability to other EMS systems remains uncertain.^4^ The score was applied to a cohort of patients transferred from a community hospital to a referral center. These patients do not resemble the more-undifferentiated population encountered by United States EMS counterparts, and the score in question was usually discussed with a stroke neurologist following arrival at the comprehensive stroke center. Although derived from the “gold standard” National Institutes of Health Stroke Severity Score, the authors acknowledged several important limitations. First, the study likely incorporated a significant amount of selection bias due to most patients being transferred from a community hospital. The RACE score was constructed from data largely obtained from patients experiencing an “anterior circulation” stroke. This component of the RACE score’s design may impact accuracy when applied to patients with middle cerebral or posterior cerebral artery circulation.^4^ The authors readily acknowledged the necessity of “larger validation studies.” To date, there has not been another published study that prospectively validates the RACE score in the context of a less-differentiated prehospital population.

Perhaps a stroke triage paradigm that emphasizes basic tenets of stroke assessment while highlighting factors linked to LVO will incentivize paramedics to make accurate triage decisions. Any optimized stroke triage protocol should incorporate additional, system-specific considerations into a comprehensive triage strategy. Factors such as focused provider education, provider level of training, and the degree of medical command oversight likely contribute to a reliable stroke protocol and assure its appropriate application to the desired patient population.

## LIMITATIONS

In this study, a statewide stroke triage protocol predicated upon a mRACE score demonstrated specificity and sensitivity inferior to previously described results. As a tool intended for the prehospital identification and triage of patients with LVO, the RACE score performed less reliably than its predecessor, the CPSS. Of course, the prehospital environment is itself somewhat chaotic, and the individual characteristics of any one system factor into the reliability of a specific triage scheme. Allegheny General Hospital provides medical oversight for numerous EMS systems within the study’s geographic area. Although the RACE exam represents an acceptable tool for EMS utilization, its implementation has been less than uniform. Provider unfamiliarity with the RACE score and the existence of different educational programs likely contribute to its variable performance.

Furthermore, providers of varied educational and certification levels (emergency medical technician [EMT], paramedic, advanced EMT) operate within the area’s EMS system. Paramedic providers more familiar with the intricacies of the central nervous system may be better positioned to formulate a diagnostic impression of stroke when compared to their BLS colleagues. In this study, paramedics were credentialed to perform and interpret the mRACE exam. BLS providers were tasked with initial stroke triage and, in some cases, requested ALS for a suspected diagnosis of LVO. This contingency represents a potential source of referral bias given that EMT providers could request ALS or critical care assistance for the treatment and transport of a possible large vessel stroke.

The mode of educational delivery highlights additional limitations. Although a state-approved program served as the foundation for instructional content, various personnel were involved in the rollout of the curriculum. Even though the EMS bureau hosted an online training program complete with case studies and triage scenarios, service agencies could incorporate their own instructors and course material into an approved mRACE program. Therefore, variations in the method of instruction and instructor familiarity with mRACE may have contributed to the score’s underperformance. Prior to the inception of the state’s mRACE protocol, providers relied upon both their clinical impression and the CPSS to arrive at a diagnosis of stroke. The study also included a subset of patients undergoing interfacility transfer. Therefore, stroke scales from outside hospitals and EMS agencies were incorporated into the patient’s medical record. Certainly, a pre-transfer diagnostic impression of stroke introduces an element of referral bias into the results. Since the protocol was an optional addition to the existing state protocols there was also relatively low penetrance among the regional EMS services.

EMS system structure varies in accordance with a system’s needs and resources This fact must be considered when choosing one stroke triage scheme over another and will likely influence the accuracy of any approved protocol. Limitations associated with retrospective chart abstraction and selection bias also represent another significant limitation. Chart abstractors were not blinded to the study hypothesis. The study hospital is one of three designated comprehensive stroke centers within the region of interest. Providers may also be more inclined to transport patients to a comprehensive stroke center knowing that the receiving hospital could treat strokes of varying severity.

Despite the use of an electronic medical record, investigators reviewed several charts with missing and incomplete data. Matching prehospital records to the inpatient electronic medical record presented additional challenges. Referral bias is another limitation that is difficult to mitigate. While the diagnosis of LVO includes specific anatomic and physiologic criteria, the absence of a clot found upon neuroimaging should not indicate a failed or inaccurate EMS referral. Indeed, a certain degree of overtriage is accepted when identifying patients who may benefit from intervention. Existing methods for validation of stroke triage fail to capture the complexity of the diagnosis. For example, an EMS provider might correctly classify a patient with post-seizure paralysis as having a LVO stroke. The particular patient’s mRACE score would be elevated due to aphasia and paralysis despite the absence of stroke-related pathology. “False positive” encounters have the potential to challenge the validity of the stroke assessment even though providers may have correctly applied the mRACE calculation. Furthermore, neurologists at comprehensive strike centers encourage transport to facilities capable of delivering the highest level of stroke care.

Distance to a comprehensive stroke center might factor into a provider’s decision to triage a sicker patient to a closer, “primary stroke” hospital. Subtleties of paramedic medical decision-making are not likely captured in our retrospective data abstraction. The diagnostic challenge of stroke presents several barriers to the responding EMS provider. Unlike other time-sensitive diagnoses such as STEMI and trauma, the diagnosis of stroke is confounded by the existence of clinical conditions that mimic stroke. The degree of stroke severity, coupled with the wide-ranging patient presentations, heap additional challenges onto the prehospital determination of LVO. Despite the Pennsylvania Bureau of EMS designating a single scale and uniform educational program for LVO triage, the mRACE score was under-represented in patient transports to a regional comprehensive stroke center.

The intervention group’s small sample size deserves mention as a significant limitation. Aside from an inability to formulate meaningful conclusions about the utility of the mRACE score, the small numbers call attention to challenges related to protocol implementation. Indeed, the process through which a “uniform” triage protocol is operationalized is exceedingly complex. Regional EMS authorities must work through problems related to provider education, training, and communications, prior to the issuance of a blanket triage protocol. Incorporation of a trial population into a stroke triage protocol might have mitigated difficulties relating to the early adoption of the mRACE score. Additional study is needed to highlight barriers to update and penetration of the LVO-specific mRACE score within the EMS system. Finally, the various stroke studies highlighted in this paper examine different clinical endpoints. It is, therefore, difficult to directly compare stroke scales since one may look exclusively at any stroke versus a LVO.

## CONCLUSION

The implementation of a novel, statewide EMS protocol intended to identify and transport patients with suspected LVO strokes performed with less than expected results. The addition of a mRACE score into existing triage protocols did not increase the sensitivity or specificity for the detection of LVO. Within a regionalized EMS service area, EMS crews reported a higher sensitivity and specificity for CPSS than the reported average. The use of mRACE for triage of LVO patients to be transported to comprehensive centers appears to perform less reliably than other prehospital triage scores such as the historically used CPSS. Prospectively oriented research is needed to better qualify the benefits of the mRACE score and other LVO- specific scores over more widely used methods. Further study is needed to identify obstacles to the prehospital detection of LVO and to inform the rollout of regional stroke triage protocols.

## Supplementary Information



## Figures and Tables

**Table 1:** Sensitivity and specificity of the Cincinatti Prehospital Stroke Scale and modified Rapid Arterial oCclusion Evaluation.

		Discharge Diagnosis		
		
		LVO	Not LVO	Total
		
CPSS	Positive	104	28	132
	Negative	23	100	123
	Total	127	128	255
mRACE	Positive	3	21	24
	Negative	1	4	5
	Total	4	25	29

*LVO*, large vessel occlusion; *CPSS*, Cincinnati Prehospital Stroke Score; *mRACE*, modified Rapid Arterial oCclusion Evaluation.

**Table 2:** Calculated sensitivity and specificity for large vessel occlusion.

	N	Sensitivity	Specificity
CPSS	255	82%	78%
mRACE	29	75%	16%

*CPSS*, Cincinnati Prehospital Stroke Score; *mRACE*, modified Rapid Arterial oCclusion Evaluation.

## References

[1] Powers WJ, Rabinstein AA, Ackerson T (2018). 2018 Guidelines for the Early Management of Patients With Acute Ischemic Stroke: A Guideline for Healthcare Professionals From the American Heart Association/American Stroke Association. Stroke.

[2] Purrucker JC, Härtig F, Richter H (2017). Design and validation of a clinical scale for prehospital stroke recognition, severity grading and prediction of large vessel occlusion: the shortened NIH Stroke Scale for emergency medical services. BMJ Open.

[3] Smith EE, Kent DM, Bulsara KR (2018). Accuracy of prediction instruments for diagnosing large vessel occlusion in individuals with suspected stroke: a systematic review for the 2018 Guidelines for the Early Management of Patients with Acute Ischemic Stroke. Stroke.

[4] Pérez de la Ossa N, Carrera D, Gorchs M (2014). Design and validation of a prehospital stroke scale to predict large arterial occlusion: the rapid arterial occlusion evaluation scale. Stroke.

[5] Schlemm L, Ebinger M, Nolte CH, Endres M (2018). Impact of prehospital triage scales to detect large vessel occlusion on resource utilization and time to treatment. Stroke.

[6] Motyer R, Kok HK, Asadi H (2017). Outcomes of endovascular treatment for acute large-vessel ischaemic stroke more than 6 h after symptom onset. J Intern Med.

[7] Saver JL, Goyal M, van der Lugt A (2016). Time to treatment with endovascular thrombectomy and outcomes from ischemic stroke: a meta-analysis. JAMA.

[8] Sequeira D, Martin-Gill C, Kesinger MR (2016). Characterizing strokes and stroke mimics transported by helicopter emergency medical services. Prehosp Emerg Care.

[9] Carrera D, Campbell BCV, Cort??s J (2017). Predictive value of modifications of the prehospital rapid arterial occlusion evaluation scale for large vessel occlusion in patients with acute stroke. J Stroke Cerebrovasc Dis.

[10] Berkhemer OA, Fransen PSS, Beumer D (2015). A randomized trial of Intra-arterial treatment for acute ischemic stroke. N Engl J Med.

[11] Goyal M, Demchuk AM, Menon BK (2015). Randomized assessment of rapid endovascular treatment of ischemic stroke. N Engl J Med.

[12] Jovin TG, Chamorro A, Cobo E (2015). Thrombectomy within 8 hours after symptom onset in ischemic stroke. N Engl J Med.

[13] Albers GW, Marks MP, Kemp S (2018). Thrombectomy for stroke at 6 to 16 hours with selection by perfusion imaging. N Engl J Med.

[14] Nogueira RG, Jadhav AP, Haussen DC (2018). Thrombectomy 6 to 24 hours after stroke with a mismatch between deficit and infarct. N Engl J Med.

[15] Liberman AL, Prabhakaran S (2017). Stroke chameleons and stroke mimics in the emergency department. Curr Neurol Neurosci Rep.

[16] Kim J-T, Chung P-W, Starkman S (2017). Field validation of the Los Angeles Motor Scale as a tool for paramedic assessment of stroke severity. Stroke.

[17] Gropen TI, Boehme A, Martin-Schild S (2018). Derivation and validation of the Emergency Medical Stroke Assessment and comparison of large vessel occlusion scales. J Stroke Cerebrovasc Dis.

[18] Turc G, Maïer B, Naggara O (2016). Clinical scales do not reliably identify acute ischemic stroke patients with large-artery occlusion. Stroke.

[19] De Luca A, Giorgi Rossi P, Villa GF, Stroke group Italian Society pre hospital emergency S (2013). The use of Cincinnati Prehospital Stroke Scale during telephone dispatch interview increases the accuracy in identifying stroke and transient ischemic attack symptoms. BMC Heal Serv Res.

[20] Turc G, Maïer B, Naggara O (2014). The use of Cincinnati Prehospital Stroke Scale during telephone dispatch interview increases the accuracy in identifying stroke and transient ischemic attack symptoms. Stroke.

[21] Jumaa MA, Castonguay AC, Salahuddin H (2019). Long-term implementation of a prehospital severity scale for EMS triage of acute stroke: a real-world experience. Neurointerv Surg.

[22] Brandler ES, Sharma M, Sinert RH, Levine SR (2014). Prehospital stroke scales in urban environments: a systematic review. Neurology.

[23] Richards CT, Huebinger R, Tataris KL (2018). Cincinnati Prehospital Stroke Scale can identify large vessel occlusion stroke. Prehosp Emerg Care.

[24] Kothari RU, Pancioli A, Liu T, Brott T, Broderick J (1999). Cincinnati Prehospital Stroke Scale: reproducibility and validity. Ann Emerg Med.

