# Supplementary material for: Challenges Related to the Implementation of an EMS-Administered, Large Vessel Occlusion Stroke Score
Source: West J Emerg Med. 2019 Oct 21;21(2):441–8. doi: 10.5811/westjem.2019.9.43127 (PMC7081843; doi:10.5811/westjem.2019.9.43127)
Supplement: Supplementary file 1 [file wjem-21-441-s001.docx]

**Appendix 1:** XX State EMS Protocol for Stroke Assessment
